# Effect of Humid Aging on the Oxygen Adsorption in SnO_2_ Gas Sensors

**DOI:** 10.3390/s18010254

**Published:** 2018-01-16

**Authors:** Koichi Suematsu, Nan Ma, Ken Watanabe, Masayoshi Yuasa, Tetsuya Kida, Kengo Shimanoe

**Affiliations:** 1 Department of Advanced Materials Science and Engineering, Faculty of Engineering Sciences, Kyushu University, Kasuga, Fukuoka 816-8580, Japan; watanabe.ken.331@m.kyushu-u.ac.jp (K.W.); yuasa@fuk.kindai.ac.jp (M.Y.); tetsuya@kumamoto-u.ac.jp (T.K.); shimanoe.kengo.695@m.kyushu-u.ac.jp (K.S.); 2 Department of Molecular and Material Science, Interdisciplinary Graduate School of Engineering Science, Kyushu University, Kasuga, Fukuoka 816-8580, Japan; manan.0611@gmail.com

**Keywords:** SnO_2_, gas sensors, humid aging, oxygen adsorption, hydroxyl poisoning, hydrogen sensing

## Abstract

To investigate the effect of aging at 580 °C in wet air (humid aging) on the oxygen adsorption on the surface of SnO_2_ particles, the electric properties and the sensor response to hydrogen in dry and humid atmospheres for SnO_2_ resistive-type gas sensors were evaluated. The electric resistance in dry and wet atmospheres at 350 °C was strongly increased by humid aging. From the results of oxygen partial pressure dependence of the electric resistance, the oxygen adsorption equilibrium constants (*K*_1_; for O^−^ adsorption, *K*_2_; for O^2−^ adsorption) were estimated on the basis of the theoretical model of oxygen adsorption. The *K*_1_ and *K*_2_ in dry and wet atmospheres at 350 °C were increased by humid aging at 580 °C, indicating an increase in the adsorption amount of both O^−^ and O^2−^. These results suggest that hydroxyl poisoning on the oxygen adsorption is suppressed by humid aging. The sensor response to hydrogen in dry and wet atmosphere at 350 °C was clearly improved by humid aging. Such an improvement of the sensor response seems to be caused by increasing the oxygen adsorption amount. Thus, the humid aging offers an effective way to improve the sensor response of SnO_2_ resistive-type gas sensors in dry and wet atmospheres.

## 1. Introduction

Resistive-type semiconductor gas sensors using SnO_2_ have been well investigated and successfully applied to commercial gas alarm systems, air quality sensors, odor sensors, and alcohol monitors for human breath since their invention 65 years ago [1,2,3,4]. SnO_2_ gas sensors detect combustible gases such as hydrogen, carbon monoxide, and ethanol by a change in the electrical resistance. The electrical resistance change is derived from the interaction of adsorbed oxygen with combustible gases at approximately 250–350 °C. The gas detection mechanism of SnO_2_ gas sensors is based on the reaction of adsorbed oxygen with combustible gases on the surface [5,6,7,8,9,10,11]. The dissociative adsorption of oxygen occurs, trapping one or two electrons from SnO_2_, as expressed by the following equations:

O_2_ + 2e^−^ → 2O^−^_ad_ (1)

O_2_ + 4e^−^ → 2O^2−^_ad_ (2)

Here, e^−^ is the carrier electron in SnO_2_, and O^−^_ad_ and O^2−^_ad_ are the adsorbed oxygen that trapped (captured) electrons. The occurrence of such oxygen adsorption was experimentally elucidated by temperature programmed desorption (TPD)-, [8] electrical resistance-, [9] and electron spin resonance (ESR)-measurements [12]. It has been well accepted that an electron depletion region is formed on the particle surface by oxygen adsorption on cation sites (Sn^4+^), [13] thereby increasing the electrical resistance [14,15]. When combustible gases are present in the gas phase, adsorbed oxygen disappears from the surface by a combustion reaction with gases and trapped electrons are transferred back to SnO_2,_ as expressed by the following equation:

H_2_ + O^−^_ad_ (or O^2−^_ad_) → H_2_O + e^−^ (or 2e^−^) (3)

Consequently, the electrical resistance of SnO_2_ decreased because of the reduction in the electron depletion region. Such a change in the electrical resistance is utilized as a signal for combustible gas detection.

Water vapor, which is always present in the atmosphere, has been recognized as the most harmful gas for semiconductor-type sensors because the adsorbed water blocks the oxygen adsorption on the surface (hydroxyl poisoning) [6,7,16,17,18]. The formation of hydroxyl groups on Sn^4+^ sites can be expressed by the following equation [16]:

H_2_O + O^−^_ad_ (or O^2−^_ad_) + 2Sn → 2(Sn-OH) +e^−^ (or 2e^−^) (4)

As mentioned above, oxygen adsorption is the key process in detecting combustible gases. Thus, surface treatment of the SnO_2_ surface is sometimes carried out to accelerate the oxygen adsorption. The surface treatment at high temperature (aging) is one way to eliminate adsorbed impurities such as by-products, water vapor, and carbonates on the particle surface. Such an aging process is important to understand the behavior of gas adsorption and reactions on the SnO_2_ surface [19,20,21]. In addition, the thermal aging is often carried out for commercial sensors to stabilize the sensor signal. However, scientific investigation of the effect of aging at high temperature on the sensor response is limited to few reports [22,23,24]. For example, Nelli et al. reported the stabilization of the sensor response of a sensor device by aging at 400 °C for 20 days [22]. Itoh et al. also proposed that long time aging in humid atmosphere improves the sensor performance in a humid atmosphere [23]. They attributed this effect to the naturalization of the particle surface by humid air at high temperature. On the other hand, Thomas et al. reported that high temperature annealing at 325 °C recovered the sensor response to hydrocarbons for a SnO_2_ semiconductor gas sensor even after its repeated use [24]. This finding implies that the aging process can refresh the particle surface to the original state. However, the annealing temperature should be sufficiently high to refresh the surface, because byproducts such as hydroxyls and carbonates that are produced by surface reaction usually desorb over 500 °C according to results on TPD measurements [6,7].

In this study, we investigated the effect of high temperature aging on the electrical and gas sensing properties of SnO_2_ semiconductor gas sensor. It is expected that high temperature aging would purify the particle surface and change the oxygen adsorption behavior. We evaluated the oxygen adsorption properties and hydrogen sensing properties after aging in humid air at 580 °C. A correlation between the electrical resistance and oxygen partial pressure was examined using a theoretical model [25]. The particular emphasis was placed on the effect of water vapor during aging treatments on oxygen adsorption properties to find a promising way that improves the gas sensing properties under humid conditions.

## 2. Materials and Methods 

### 2.1. Preparation and Characterization of SnO_2_ Nanoparticles and Sensor Fabrication

**Figure 1:**
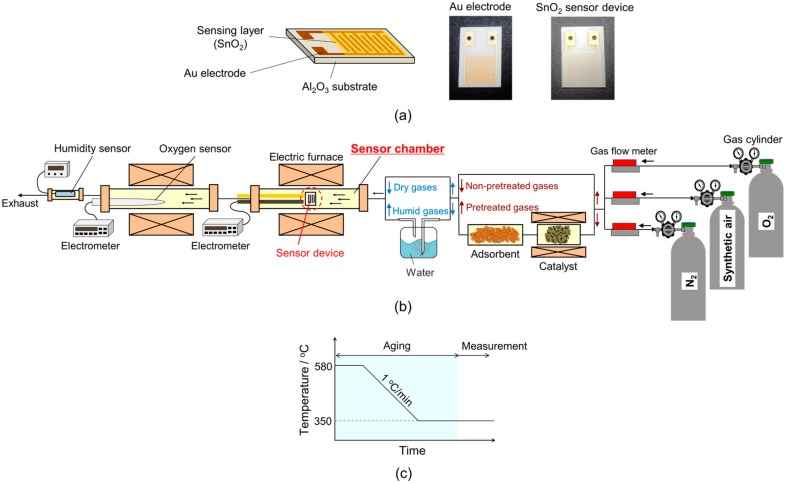
(**a**) Schematic image and photographs of the Al_2_O_3_ substrate printed with Au electrode and SnO_2_ sensor device. (**b**) Schematic drawing of the measurement apparatus and sensor device for gas sensors. (**c**) Temperature profile of the aging process.

SnO_2_ nanoparticles were synthesized by hydrothermal synthesis, as described in a previous report [9]. Stannic acid gel (SnO_2_·*n*H_2_O), which is obtained by mixing a SnCl_4_∙5H_2_O (Wako Pure Chemical Industries, Ltd., Osaka, Japan) solution (1 M) into NH_4_HCO_3_ (Kishida Chemical Company, Ltd., Osaka, Japan) solution (1 M), was hydrothermally treated at 200 °C for 3 h using an autoclave system (TAS-05 type; TAIATSU TECHNO, Tokyo, Japan) after Cl^−^ ions were removed and the solution pH was adjusted to 10.5. Then, the obtained SnO_2_ dispersed sol was dried in air at 120 °C for a half day, and then calcined at 600 °C for 3 h, producing a SnO_2_ powder. A paste containing the SnO_2_ powder in an organic binder (α-terpineol; Wako Pure Chemical Industries, Ltd.) was coated on an Al_2_O_3_ substrate (9 mm × 13 mm × 0.38 mm; Japan Fine Ceramics Company, Ltd., Miyagi, Japan) equipped with comb type Au electrodes (electrode span is 90 μm, sensing layer area is 64 mm^2^) by a screen printing method to fabricate a thick film-type sensor device. Then, the device was heated at 580 °C for 3 h in air to remove α-terpineol. The schematic image and photographs of the Au electrode and sensor device are shown in Figure 1a.

The crystal structure of the SnO_2_ particles was determined by X-ray diffractometory with copper Kα radiation (XRD; RINT 2100, RIGAKU, Tokyo, Japan), and the crystallite size was estimated from the Sherrer’s equation. The specific surface area of SnO_2_ particles was measured using a specific surface area analyzer (BELSORP-mini II; Bel Japan, Osaka, Japan). The obtained nanoparticles were observed by field-emission scanning electron microscopy (FE-SEM; S-4800, Hitachi, Tokyo, Japan).

### 2.2. Evaluation of the Electrical Properties

The electrical resistance was measured with an apparatus equipped with a gas pretreatment system, a water vapor introduction system, an oxygen sensor, and a humidity sensor, as schematically described in Figure 1b. The gas pretreatment system was equipped with a catalyst and an adsorbent of Pt (5 wt %)-loaded Al_2_O_3_ (Wako Pure Chemical Industries, Ltd.) and molecular sieves (5A 1/16, Wako Pure Chemical Industries Ltd.), respectively [9,26]. The catalyst was heated at 500 °C to remove impurities from commercial nitrogen and air. Water vapor was introduced through a bubbler containing de-ionized water, and the humidity was controlled by mixing dry and humid gases. The exact P_O2_ and humidity (partial pressure of H_2_O; P_H2O_) in the gas atmosphere were measured with a homemade oxygen sensor based on calcium stabilized zirconia (CSZ; Suzuki rikagaku Co., Ltd., Okegawa, Japan) and a commercial capacitance-type humidity sensor (TR-77Ui; T&D Corporation, Matsumoto, Japan), respectively. Temperature around the sensor device was monitored by a thermocouple that was positioned close to the sensor device. The gas flow rates of air and sample gases were adjusted to 80 cm^3^/min with mass flow controllers (SEC-series; HORIBA STEC, Kyoto, Japan).

The temperature profile of thermal aging of devices is shown in Figure 1c. Thermal aging was carried out at 580 °C for 3 h in air with different humidity levels. Then, the device was cooled down to 350 °C and the electrical resistance was measured after the resistance had stabilized. For the measurements, P_O2_ was adjusted by mixing nitrogen, synthetic air or oxygen. These gases were purified with the gas pretreatment system. Sample gases containing hydrogen in air were prepared by diluting parent synthetic gas mixture with purified synthetic air. In this case, parent synthetic gas mixtures containing hydrogen were not treated with the gas pretreatment system. The sensor device was connected with a standard resistor, and voltage across the standard resistor was measured under an applied voltage of DC 4 V to evaluate the electrical resistance of the device. The electrical signal of the sensor was acquired with an electrometer (Model 12002; Keithley Instruments, Cleveland, OH, USA), and the relative resistance and sensor response to hydrogen were defined as R/R_N2_ and R_a_/R_g_, respectively. Here, R, R_N2_, R_a_ and R_g_ are the electrical resistance in various P_O2_, nitrogen, synthetic air and sample gases, respectively.

## 3. Results and Discussion

### 3.1. Effect of Humid Aging on the Material Characteristics

**Figure 2:**
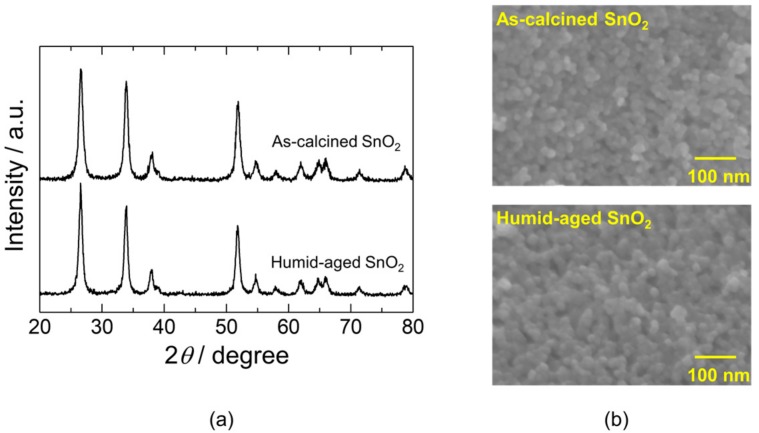
(**a**) XRD patterns and (**b**) SEM images of as-calcined and humid-aged SnO_2_ nanoparticles.

Figure 2a shows the XRD patterns of SnO_2_ nanoparticles calcined at 600 °C in air with and without aging in humified oxygen (P_H2O_: 0.04 atm (96 RH% at 30 °C)) at 580 °C for 3 h (humid aging). These XRD patterns were well matched with those of cassiterite stannic acid (JCPDS 41-1445). No impurity phases were observed. The estimated crystallite sizes of SnO_2_ nanoparticles without and with humid aging were 12 and 14 nm, respectively. The SnO_2_ nanoparticles slightly grew up after the humid aging. However, no further crystal growth was not observed even after repeated humid aging. Specific surface areas of the SnO_2_ nanoparticles without and with the humid aging were 27 and 24 m^2^·g^−1^, respectively. No significant change in the surface area was observed after the humid aging. The SEM images of the SnO_2_ nanoparticles confirmed no obvious difference in morphology after the humid aging, as shown in Figure 2b. The above results conclude that the humid aging slightly facilitated the growth of SnO_2_ crystals, consequently reducing their specific surface area.

### 3.2. Effect of Humid Aging on the Oxygen Adsorption

**Figure 3:**
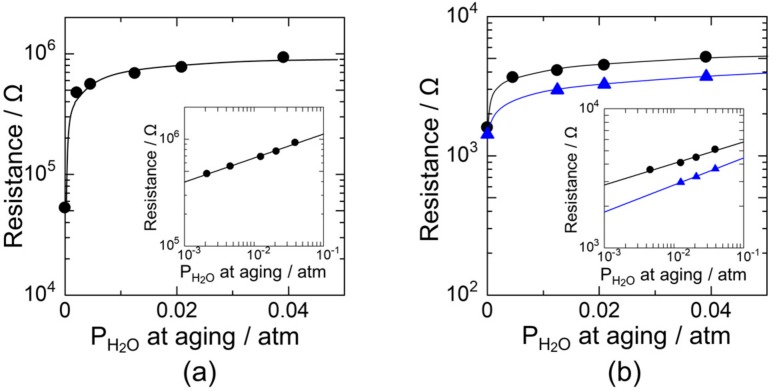
The electrical resistance of SnO_2_ nanoparticles in (**a**) dry and (**b**) wet air atmosphere at 350 °C as a function of P_H2O_ in humid aging. P_H2O_ in wet air atmosphere are 0.005 and 0.01 atm.

Figure 3a shows the electrical resistance at 350 °C in dry air of the device that was aged at 580 °C in humid atmosphere (humid aging) as a function of humidity at the humid aging. Here, P_O2_ at the aging process was kept at 0.21 atm (synthetic air) and P_H2O_ at the aging process was controlled from 0.039 atm (93 RH% at 30 °C) to 0.002 atm (6.4 RH% at 25 °C). Aging was also carried out in dry air at 580 °C (dry aging). The electrical resistance at 350 °C in dry air drastically increased after introducing water vapor during the aging processes. The larger the humidity at the aging process at 580 °C, the larger the electrical resistance in air at 350 °C was, as shown in the inset of Figure 3a. Generally, the presence of the humidity decreases the electrical resistance of SnO_2_ gas sensors due to the hydroxyl poisoning on the particle surface, blocking the oxygen adsorption and decreasing the resistance. [16] In contrast, the tendency observed here is opposite to that reported as the hydroxyl poisoning effect. The resistance at 350 °C increased in dry air after the humid aging. The results here clearly indicate that the humid aging at 580 °C promoted the oxygen adsorption at 350 °C. According to some previous reports, high temperature aging can remove adsorbed gases from the particle surface. For example, a TPD analysis shows that desorption of adsorbed hydroxyls occurs at temperatures higher than 300 °C [6,7]. Thus, it is possible that hydroxyl groups desorbed from the SnO_2_ surface at 350 °C, assisting in the adsorption of oxygen on the surface and increasing the electrical resistance.

We also measured the electrical resistance at 350 °C in wet air after the humid aging at 580 °C, as shown in Figure 3b. Here, the humidity in wet air at 350 °C was set to 0.01 atm (32 RH% at 25 °C) and 0.005 atm (16 RH% at 25 °C). The electrical resistance in wet air was much lower than that in dry air. The electrical resistance at 350 °C was 5.3 × 10^4^ to 9.3 × 10^5^ Ω in dry air, but it decreased to 1.6–5.0 × 10^3^ Ω in wet air (P_H2O_: 0.005 atm) even after the humid aging, as shown in Figure 3a,b. This indicates that the hydroxyl poisoning occurred to the device. However, it is noteworthy that increasing the humidity during the humid aging increased the electrical resistance in wet air at 350 °C. This phenomenon suggests that humid aging can weaken the hydroxyl poisoning effect.

Recently, the relationship between the electrical resistance and P_O2_ has been theoretically proposed as the following equation [25]:

(5) $$\frac{R}{R_{0}}\;=\;\frac{1}{2}\left(c+\frac{3}{a}K_{1}{}^{\frac{1}{2}}P_{O_{2}}{}^{\frac{1}{2}}\right)+{\left\{\frac{1}{4}{\left(c+\frac{3}{a}K_{1}{}^{\frac{1}{2}}P_{O_{2}}{}^{\frac{1}{2}}\right)}^{2}+\frac{6N_{D}}{a}\cdot K_{2}{}^{\frac{1}{2}}P_{O_{2}}{}^{\frac{1}{2}}\right\}}^{\frac{1}{2}}$$

Here, R/R_0_ is the relative resistance, R and R_0_ are the electrical resistance in the atmosphere with and without oxygen, respectively, *a* is the crystallite radius, *c* is the constant value, *N_D_* is the donor density, and P_O2_ is the oxygen partial pressure. *K*_1_ and *K*_2_ are equilibrium constants, which are the ratio of the rate constants of the forward and reverse reactions of the Equations (1) and (2), respectively. Here, if *K*_1_ or *K*_2_ is zero, Equation (5) can be more simplified as the following equations, respectively.

(6) $$\frac{R}{R_{0}}\;=\;c+\frac{3}{a}K_{1}{}^{\frac{1}{2}}P_{O_{2}}{}^{\frac{1}{2}}\;(O^{-}\mathrm{formation},K_{2}=0)$$

(7) $$\frac{R}{R_{0}}\;=\;{\left\{\frac{1}{4}c^{2}+\frac{6N_{D}}{a}\cdot K_{2}{}^{\frac{1}{2}}P_{O_{2}}{}^{\frac{1}{2}}\right\}}^{\frac{1}{2}}\;(O^{2-}\mathrm{formation},K_{2}=0)$$

Recently, we have evaluated the oxygen adsorption species by the linear relationship between the electrical resistance and P_O2_^1/2^ or P_O2_^1/4^ using the Equations (6) and (7), respectively. According to our previous report, oxygen dissociatively adsorbed as O^2−^ on the SnO_2_ surface at 350 °C in dry atmosphere. The adsorption of O^2−^ is supported by the fact that the electrical resistance is proportional to the P_O2_^1/4^ [9,27]. However, there is a possibility that the adsorption of O^−^ and O^2−^ occur competitively. Thus, in this study, we tried to estimate the equilibrium constants (oxygen adsorption ability) of O^−^ and O^2−^ adsorption using the Equation (5) by examining the correlation between the relative resistance and P_O2_. We expected that humid aging affects the oxygen adsorption properties, leading to a change in *K*_1_ and *K*_2_ values.

**Figure 4:**
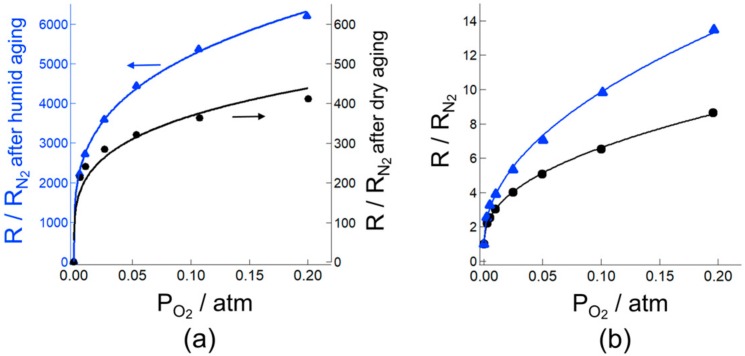
(**a**) The relative resistance (R/R_N2_) at 350 °C in (**a**) dry and (**b**) humid (P_H2O_; 0.012 atm) atmosphere as a function of oxygen partial pressure after (●) dry and (▲) humid aging.

The relative resistance, (R/R_N2_), at 350 °C after the dry and humid aging was plotted as a function of P_O2_ are shown in Figure 4a. We used the electrical resistance in N_2_ (R_N2_) in wet atmosphere in place of the resistance without oxygen (R_0_) because complete removal of oxygen from nitrogen is experimentally difficult. The nitrogen gas used in this study contained approximately 50 ppm oxygen. This residual oxygen has a strong influence on R_N2_. For example, R_N2_ values in dry and wet atmospheres were 3.7 × 10^3^ and 1.3 × 10^2^ Ω, respectively. Difference of these electrical resistances was caused by the oxygen adsorption amount because hydroxyl poisoning disturbs the oxygen adsorption. The lower electrical resistance in wet atmosphere indicate that the adsorption of residual oxygen in N_2_ was blocked by humidity. Thus, R_N2_ in wet atmosphere seems to be closer to the value of R_0_ than R_N2_ in dry atmosphere. Because of this reason, we used R_N2_ in wet atmosphere in place of R_0_. Here, P_H2O_ in the humid aging was controlled at 0.04 atm (96 RH% at 30 °C). Aging was also carried out in dry air (0 RH%, dry aging). The relative resistance clearly increased by the humid aging, as shown in Figure 4a. It should be noted that the relative resistance corresponds to the sensitivity to oxygen. Thus, the increase in the relative resistance indicates that the amount of oxygen adsorption increased after the humid aging. The results were in perfect fit with Equation (5), as shown in Figure 4a. Using the fitting results, we estimated the oxygen adsorption equilibrium constants for O^−^ adsorption (*K*_1_) and O^2−^ adsorption (*K*_2_) after the dry or humid aging. Here, the donor density of SnO_2_ was set to 5.0 × 10^18^ cm^−3^, as reported in literature [15]. Dimensions of *K*_1_ and *K*_2_ can be derived from Equations (1) and (2), respectively, when dimensions of the surface oxygen concentration [O^−^] (or [O^2−^]) and the electron density [e^−^] were cm^−2^ and cm^−3^, respectively. The estimated *K*_1_ and *K*_2_ values are summarized in Table 1. Absolute *K*_1_ and *K*_2_ values after the humid aging are about four and five orders larger than those after the dry aging. Increases in *K*_1_ and *K*_2_ indicate the increase in the adsorbed oxygen amount. Thus, the obtained results conclude that the humid aging increased the amount of adsorbed oxygen as both forms of O^−^ and O^2−^.

**Figure 5:**
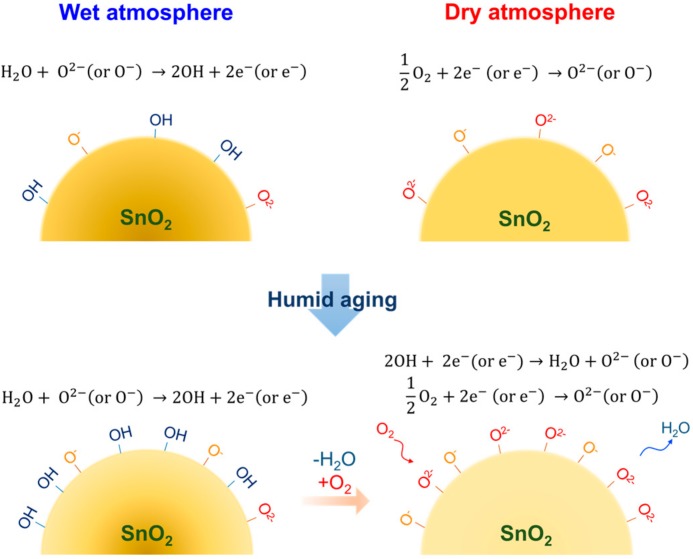
Schematic model of the effect of humid aging for oxygen adsorption on the SnO_2_ surface in dry and wet atmospheres.

The oxygen adsorption behaviors in wet air at 350 °C after humid aging were also studied. Figure 4b shows the relative resistance at 350 °C in wet air (P_H2O_: 0.012 atm, 38 RH% at 25 °C) after the dry and humid aging. Humid aging was carried out in air containing 0.05 atm H_2_O (100 RH% at 33 °C). Aging was also carried out in dry air. The relative resistance in wet air increased after the humid aging. This trend is in consistent with the case for the relative resistance measurements in dry air (Figure 4a). However, the absolute value of the relative resistance in wet air is significantly smaller than that in dry air, suggesting that hydroxyl poisoning occurred in wet air. Nevertheless, the results were also well fitted with Equation (5), as shown in Figure 4b. The estimated *K*_1_ and *K*_2_ values are summarized in Table 2. The *K*_1_ value (4 × 10^−11^ cm^2^∙atm^−1^) in wet air after humid aging is larger than that (1 × 10^−11^ cm^2^∙atm^−1^) after dry aging, indicating that the amount of the O^−^ adsorption was increased by the humid aging. In contrast, the *K*_2_ value in wet air after the humid aging is smaller than that after dry aging. The results suggest that hydroxyl poisoning was more significant in the O^2−^ adsorption. The significant decrease in *K*_2_ (7 × 10^−50^ cm^8^∙atm^−1^) in wet air is also prominent by comparing the values with those (3 × 10^−49^ cm^8^∙atm^−1^) in dry air (Table 1), suggesting that the O^−^ adsorption became predominant in wet air. In the previous report, we demonstrated that the main oxygen adsorption specie is transferred from O^2−^ to O^−^ by introducing water vapor in a measurement atmosphere [27]. Thus, the observed changes in *K*_1_ and *K*_2_ are in good agreement with the previous results. Recently, Watanabe et al. reported that exchanges of surface oxygen with atmospheric oxygen efficiently proceeds on indium-gallium-zinc oxide in the presence of humidity [28]. On the basis of their work, it might be possible that the oxygen adsorption on the SnO_2_ surface proceeds via oxygen exchange at hydroxyl groups that were formed at high temperature during humid aging. Hence, humid aging leads increasing the amount of oxygen adsorption on the SnO_2_ than dry aging. The proposed oxygen adsorption model was schematically shown in Figure 5. The surface naturalization by humid aging, which is empirically known as an effective way to improve the sensor response in practical gas sensors, may also contribute to the increase in the amount of oxygen adsorption. However, unfortunately, no experimental evidences to support these ideas have been obtained yet. Nevertheless, we can conclude that the humid aging is one of the efficient approaches to enhance the oxygen adsorption on the SnO_2_ surface even in humid atmospheres.

### 3.3. Effects of Humid Aging on the Sensor Response to Hydrogen

The sensor response to the combustible gases (S = R_a_/R_g_) is defined as the ratio of the electrical resistance in air (R_a_) to that in air containing combustible gases (R_g_). The electrical resistance in air is used as a base line. According to this definition, an increase in R_a_ should result in an increase in the sensor response. Hence, we expected that the sensor response would be enhanced by humid aging that suppresses hydroxyl poisoning of oxygen adsorption.

**Figure 6:**
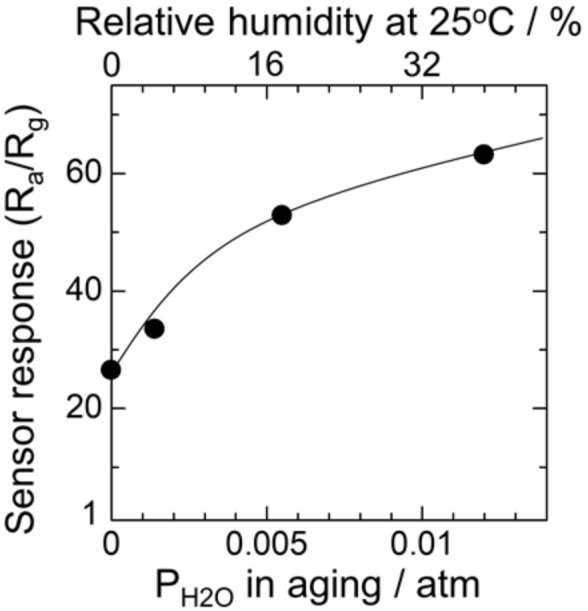
The sensor response to 200 ppm hydrogen at 350 °C in dry atmosphere as a function of P_H2O_ in aging.

Figure 6 shows the sensor response to 200 ppm hydrogen in dry air at 350 °C as a function of P_H2O_ during aging for the device with humid aging at 580 °C. The sensor response was improved by the humid aging with higher humidities, as expected above. Thus, humid aging is effective in enhancing the sensor response to hydrogen because of the promotion of the oxygen adsorption.

**Figure 7:**
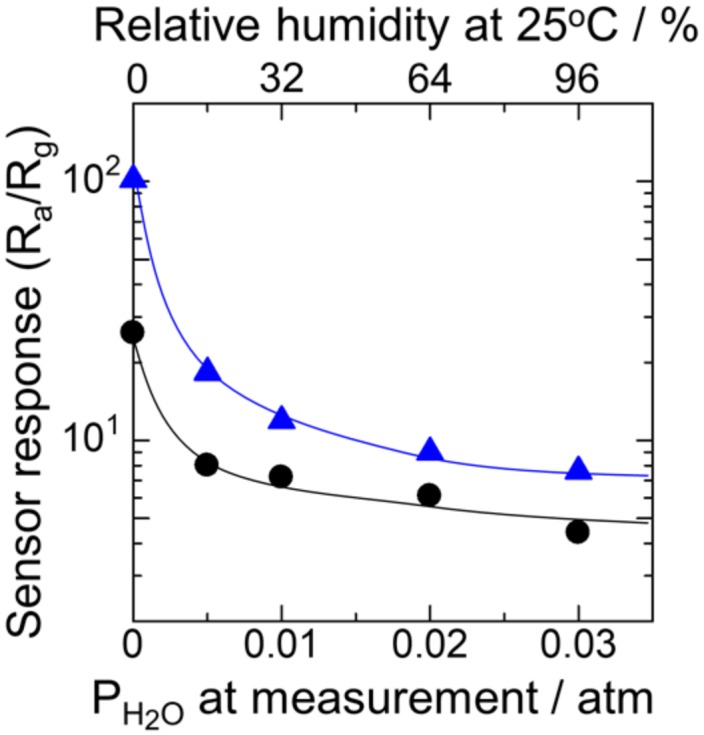
Relationships between the sensor response to 200 ppm H_2_ at 350 °C as a function of P_H2O_ in measurement after (●) dry and (▲) humid aging (P_H2O_ is 0.03 atm).

Next, the sensor response in wet air was examined for the device with humid aging (P_H2O_: 0.03 atm, 96 RH% at 25 °C) at 580 °C. Figure 7 shows the sensor responses to hydrogen as a function of humidity in sample gases (P_H2O_: 0–0.03 atm) at 350 °C. The sensor response was significantly deteriorated by humidity for the device with the dry aging. In contrast, such a significant decrease in the sensor response was suppressed after the humid aging. As shown in Figure 4b, we observed that humid aging enhanced the oxygen adsorption. Thus, an increase in the amount of oxygen on the surface by the humid aging facilitated the combustion of hydrogen, thereby suppressing the hydroxyl poisoning [16,17,18]. However, in high wet air (P_H2O_: 0.03 atm), the sensor response became less than S = 10. Hence, further modification of the surface conditions is necessary. Thus, the results demonstrate that humid aging is effective in improving the sensor response in wet atmospheres, especially at 350 °C.

## 4. Conclusions

In this study, we investigated the effect of aging at 580 °C in wet air (humid aging) on the oxygen adsorption and gas sensing properties for SnO_2_ resistive-type gas sensors. We found that humid aging slightly increased the crystallite size, leading to a slight decrease in the specific surface area of SnO_2_ nanoparticles. However, no significant difference in morphology was seen by SEM observations before and after the humid aging. In contrast, the humid aging strongly affected the electrical properties of the SnO_2_ nanoparticles. The electrical resistance in air was drastically increased by the humid aging process. By analyzing the relationship between the resistance and P_O2_, we revealed increases of the oxygen adsorption equilibrium constants (*K*_1_ and *K*_2_) after humid aging. These increases in *K*_1_ and *K*_2_ appeared not only in a dry atmosphere but also in a humid atmosphere. Thus, hydroxyl poisoning was suppressed by humid aging that increases the amount of oxygen adsorption. Consequently, the sensor response to hydrogen was improved by the humid aging. Such an improvement in the sensor response was also confirmed even in a wet atmosphere. Therefore, we conclude that humid aging is an efficient way to enhance the oxygen adsorption on the SnO_2_ surface and consequently improve the sensor response to combustible gases in dry and wet atmospheres. We also believe that this finding is important in fundamental understanding on the influence of humidity for practical gas sensors.

## Figures and Tables

**Table 1:** Estimated equilibrium constants of oxygen adsorption for O^−^ (*K*_1_) and O^2−^ (*K*_2_) at 350 °C in dry air. The device was aged under dry and humid conditions.

P_H2O_ in Aging Atmosphere/atm	Measurement Atmosphere	*K*_1_ (cm^2^∙atm^−1^)	*K*_2_ (cm^8^∙atm^−1^)
dry	dry	8 × 10^−11^	8 × 10^−41^
0.04 (wet)	dry	5 × 10^−7^	3 × 10^−36^

**Table 2:** Estimated equilibrium constants of oxygen adsorption for O^−^ (*K*_1_) and O^2−^ (*K*_2_) at 350 °C in wet air. The device was aged under dry and humid conditions.

P_H2O_ in Aging Atmosphere/atm	Measurement Atmosphere	*K*_1_/cm^2^∙atm^−1^	*K*_2_/cm^8^∙atm^−1^
dry	0.012 (wet)	1 × 10^−11^	3 × 10^−49^
0.05 (wet)	0.012 (wet)	4 × 10^−11^	7 × 10^−50^
